# Sulforaphane Represses Matrix-Degrading Proteases and Protects Cartilage From Destruction In Vitro and In Vivo

**DOI:** 10.1002/art.38133

**Published:** 2013-01-01

**Authors:** Rose K Davidson, Orla Jupp, Rachel de Ferrars, Colin D Kay, Kirsty L Culley, Rosemary Norton, Clare Driscoll, Tonia L Vincent, Simon T Donell, Yongping Bao, Ian M Clark

**Affiliations:** 1 University of East AngliaNorwich, UK; 2 Kennedy Institute of RheumatologyLondon, UK; 4 University of OxfordOxford, UK; 3 Norfolk and Norwich University HospitalNorfolk, UK

## Abstract

**Objective:**

Sulforaphane (SFN) has been reported to regulate signaling pathways relevant to chronic diseases. The aim of this study was to investigate the impact of SFN treatment on signaling pathways in chondrocytes and to determine whether sulforaphane could block cartilage destruction in osteoarthritis.

**Methods:**

Gene expression, histone acetylation, and signaling of the transcription factors NF-E2–related factor 2 (Nrf2) and NF-κB were examined in vitro. The bovine nasal cartilage explant model and the destabilization of the medial meniscus (DMM) model of osteoarthritis in the mouse were used to assess chondroprotection at the tissue and whole-animal levels.

**Results:**

SFN inhibited cytokine-induced metalloproteinase expression in primary human articular chondrocytes and in fibroblast-like synovial cells. SFN acted independently of Nrf2 and histone deacetylase activity to regulate metalloproteinase expression in human articular chondrocytes but did mediate prolonged activation of JNK and p38 MAPK. SFN attenuated NF-κB signaling at least through inhibition of DNA binding in human articular chondrocytes, with decreased expression of several NF-κB–dependent genes. Compared with cytokines alone, SFN (10 μ*M*) abrogated cytokine-induced destruction of bovine nasal cartilage at both the proteoglycan and collagen breakdown levels. An SFN-rich diet (3 μmoles/day SFN versus control chow) decreased the arthritis score in the DMM model of osteoarthritis in the mouse, with a concurrent block of early DMM-induced gene expression changes.

**Conclusion:**

SFN inhibits the expression of key metalloproteinases implicated in osteoarthritis, independently of Nrf2, and blocks inflammation at the level of NF-κB to protect against cartilage destruction in vitro and in vivo.

Two key molecules that endow cartilage extracellular matrix with its structural properties are type II collagen and the proteoglycan aggrecan. The former molecule is principally turned over by the action of collagenolytic matrix metalloproteinases (MMPs; e.g., MMP-1 and MMP-13), while enzymes from the ADAMTS family are responsible for metabolism of the latter molecule (1). An imbalance between the activity of key enzymes from these families and their inhibitors is thought to underlie cartilage destruction in osteoarthritis (OA).

Epidemiology data suggest that high intake of fruit and vegetables may protect against the onset and/or progression of OA (2–4). Sulforaphane (1-isothiocyanato-4-methylsulphinylbutane; SFN) is a plant-derived isothiocyanate obtained in the diet through consumption of cruciferous vegetables, particularly broccoli (5). SFN is a potent inducer of phase II (detoxification) metabolism via activation of the transcription factor NF-E2–related factor 2 (Nrf2), which binds to an antioxidant response element in cognate genes (5,6). SFN can impact on several signaling pathways in a cell type–dependent manner. The antiinflammatory properties of SFN have been reported previously (7–9), and these effects were suggested to function through NF-κB, activator protein 1, and MAPK signaling. Modulation of MMP expression in chondrocytes by SFN has been previously described (9–11). The efficacy of SFN (at high doses) in protecting mice with experimentally induced inflammatory arthritis has been demonstrated, and in vitro experiments using T cells from patients with rheumatoid arthritis showed a reduction in the activation and production of interleukin-17 (IL-17) and tumor necrosis factor α (TNFα) (12). Epigenetic regulation by SFN has also been reported in vitro and in vivo (13). We previously showed that broad-spectrum histone deacetylase (HDAC) inhibitors are chondroprotective agents (14), in part via repression of MMP expression, and this finding was supported in animal models of arthritis (15,16). In the current study, we sought to determine the efficacy of SFN, which has been reported as a weak HDAC inhibitor (17), as a chondroprotective agent.

## MATERIALS AND METHODS

### Materials

SFN and its metabolites were obtained from Toronto Research Chemicals, except SFN–Cys-Gly, which was synthesized by Dr. Sunil Sharma, University of East Anglia. IL-1 and oncostatin M (OSM) were obtained from R&D Systems. NF-κB p65 (catalog no. sc-109 X and no. sc-372), p50, and c-Rel primary antibodies were obtained from Santa Cruz Biotechnology. All other primary antibodies (phospho-p65; catalog no. 3033), IκBα (catalog no. 4814S), acetylated histone H3 (catalog no. 4353S), histone H3 (catalog no. 9715S), acetylated Lys (catalog no. 9441S), GAPDH (catalog no. 2118S), JNK (catalog no. 9258S), ERK (catalog no. 9102), p38 (catalog no. 9212), phospho-JNK (catalog no. 4668S), phospho-ERK (catalog no. 9101S), and phospho-p38 (catalog no. 4511S) were from Cell Signaling Technology. Small interfering RNA (siRNA) against Nrf2 (Ambion) and AllStars nontargeting siRNA were from Qiagen; staurosporine was obtained from Sigma-Aldrich; and trichostatin A and sodium butyrate were from Calbiochem. NF-κB consensus sequence IRDye 700–labeled oligos were from Li-Cor. The IκBα promoter reporter plasmid was a gift from Prof. Derek Mann, Newcastle University, UK (originally from Prof. Ronald Hay, University of Dundee, UK).

### Cell culture and treatments

The SW-1353 human chondrosarcoma cell line was purchased from ATCC. Primary human articular chondrocytes were isolated from the cartilage of patients with OA who underwent knee replacement surgery, as previously described (16). All human articular chondrocytes were used at passages 1–2. Fibroblast-like synovial cells (FLS) were cultured from the synovial tissue of patients with OA, with tissue dissected into ∼1-cm^3^ pieces and placed in culture to allow cell outgrowth. These cells were seeded for the experiments, as described. This study was performed with ethics approval (Norfolk Ethics Committee), and all patients provided informed consent.

Cells were cultured in Dulbecco’s modified Eagle’s medium (DMEM; GlutaMAX) supplemented with 10% fetal calf serum volume/volume, 1,000 IU/ml penicillin, and 100 μg/ml streptomycin at 37°C in an atmosphere of 5% CO_2_. Cells were plated at 1.2 × 10^4^ cells/cm^2^, left to adhere overnight, and serum-starved overnight prior to treatment. Cells or cartilage tissue specimens were preincubated with SFN for 30 minutes prior to cytokine stimulation.

### Complementary DNA (cDNA) synthesis and quantitative reverse transcription–polymerase chain reaction (qRT-PCR)

Whole cell lysates were harvested into 30 μl of Cells-to-cDNA II Cell Lysis Buffer (Ambion). Lysates (8 μl) treated with DNase I (Ambion) were reverse transcribed in a total volume of 20 μl, using 200 ng random primers and 100 units Moloney murine leukemia virus reverse transcriptase (Invitrogen), according to the manufacturer’s instructions, in the presence of 40 units RNasin (Promega).

Relative quantification of genes was performed using an ABI Prism 7500 Sequence Detection System (Applied Biosystems). PCRs used 5 μl of reverse-transcribed RNA (a 10-fold dilution of cDNA was used for 18S analyses). The *MMP* and *ADAMTS* primers and probes were previously described (18,19). The primers and probes for *IL8*, *IL6*, *INOS*, *Nrf2*, *A20*, *IκBα*, *COX2*, *SOD2*, and *HMOX1* were designed using the Universal Probe Library (Roche). Relative quantification is expressed as 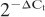, and all data were normalized to 18S ribosomal RNA expression.

Total RNA was extracted and purified from whole mouse joints, using TRIzol reagent (Invitrogen) according to the manufacturer’s instructions. RNA quality was analyzed using an Agilent 2100 Bioanalyzer. Sample replicates were pooled and hybridized to an Illumina Mouse WG-6 whole-genome array (Source BioScience). Probe signal underwent quantile normalization, and messenger RNA (mRNA) levels were validated by qRT-PCR in replicates.

### Gene silencing

Human articular chondrocytes were transfected using DharmaFECT 1 (Thermo Scientific) with 25 n*M* siRNA against Nrf2 or nontargeting AllStars siRNA (Qiagen) for 24 hours prior to SFN and cytokine treatments. All treatments were carried out in quadruplicate. Gene expression was measured using qRT-PCR.

### Western blotting

Whole cell lysates were harvested into ice-cold radioimmunoprecipitation assay buffer (50 m*M* Tris HCl, pH 7.6, 150 m*M* NaCl, 1% v/v Triton X-100, 1% weight/volume sodium deoxycholate, 0.1% w/v sodium dodecyl sulfate, 10 m*M* NaF, 2 m*M* Na_3_VO_4_, 1× protease inhibitor cocktail [Fisher Scientific]). Cytosolic and nuclear cell fractions were obtained by adding 500 μl hypotonic buffer (20 m*M* Tris HCl, pH 7.4, 10 m*M* NaCl, 3 m*M* MgCl_2_) to cell pellets and incubated for 15 minutes on ice. Nonidet P40 (NP40; 25 μl 10% v/v) was added and vortexed for 10 seconds. Samples were centrifuged for 10 minutes at 300*g*. Supernatant was collected (cytosolic fraction) and stored at −20°C. Fifty microliters of nuclear extraction buffer (100 m*M* Tris HCl, pH 7.4, 100 m*M* NaCl, 1% v/v Triton X-100, 1 m*M* EDTA, 1 m*M* EGTA, 10% v/v glycerol, 0.1% w/v sodium dodecyl sulfate [SDS], 0.5% w/v deoxycholate, 1× protease inhibitor cocktail, and phosphatase inhibitors) was added to the pellets and incubated for 30 minutes on ice, with vortexing every 10 minutes. Samples were centrifuged at 14,000*g* for 3 minutes at 4°C. Supernatants were stored at −80°C. Samples were separated on reducing SDS–polyacrylamide gel electrophoresis gels, transferred to PVDF membranes, and probed overnight at 4°C. Proteins were detected using horseradish peroxidase–conjugated secondary antibodies (Dako). Bands were visualized using LumiGLO reagent (New England Biolabs) and exposure to Kodak BioMax MS film (Sigma-Aldrich).

### Immunocytochemical analysis

Human articular chondrocytes were grown on chamber slides at a density of 3.75 × 10^4^/cm^2^ and treated with SFN (10 μ*M*) for 30 minutes prior to stimulation with IL-1 (5 ng/ml) for 45 minutes. Cells were probed for NF-κB p65 rabbit polyclonal antibody (Santa Cruz Biotechnology) at 1:100 dilution, followed by the secondary antibody, Cy3-conjugated goat anti-rabbit IgG (Abcam) at 1:200 dilution. Nuclei were stained with DAPI and examined using a Zeiss AxioPlan 2IE fluorescence microscope at 20× magnification. Negative controls omitted the primary antibody. Images were acquired and analyzed with AxioVision version 4.7 software.

### Electrophoretic mobility shift assay (Emsa)

For the preparation of nuclear extracts, cells were lysed in 0.1% v/v NP40 in phosphate buffered saline on ice for 1 minute, and then centrifuged. Thereafter, the pellets were suspended in 3× volume high-salt buffer (25 m*M* HEPES, pH 7.8, 500 m*M* KCl, 0.5 m*M* MgSO_4_, 1 m*M* dithiothreitol [DTT], protease, and phosphatase inhibitors]), and incubated for 20 minutes on ice, with occasional mixing. Samples were centrifuged at high speed for 2 minutes, and supernatant was stored at −80°C. Protein was quantified using Bradford Reagent (Bio-Rad). Nuclear extracts were analyzed for DNA binding using the Li-Cor protocol for NF-κB IRDye 700–labeled oligos. Nuclear extracts containing 5 μg total protein were added to the binding reactions at room temperature for 20 minutes in the dark. DNA binding was visualized using an Odyssey infrared imaging system (Li-Cor). The NF-κB consensus sequence (mutant G/C) was 5′-AGTTGAGGG/CGACTTTCCCAGGC-3′.

### Transfection and gene promoter reporter assay

SW-1353 cells were plated at 2 × 10^4^/well in a 24-well plate and left to adhere. Transfections were carried out using 200 ng plasmid DNA and 0.5 μl Lipofectamine 2000 (Fisher Scientific) for 24 hours. The culture medium was changed to serum-free overnight, after which the cells were treated for 6 hours. Fifty microliters of luciferin substrate (Promega) was added to 10 μl cell lysate, and luminescence was measured immediately using an EnVision Multilabel Plate Reader (PerkinElmer).

### High-performance liquid chromatography tandem mass spectrometry (HPLC-MS/MS) analysis

Primary chondrocytes or SW-1353 cells were seeded at a density of 1.7 × 10^4^/cm^2^ and grown to confluence. Medium was replaced with phenol-free/serum-free DMEM containing 10 μ*M* SFN and incubated for 0–2 hours. Samples were acidified with formic acid, and the internal standard iberin (10 μ*M*) was added.

HPLC-MS/MS analysis was carried out using an Agilent 1200 Series HPLC System linked to an AB Sciex Q-Trap 3200 MS/MS system. Separation was performed using a Kinetex pentafluorophenyl reverse-phase HPLC column (2.6 μm, 100 × 4.60 mm; Phenomenex) at 37°C. The flow rate was 1 ml/minute, using 0.1% v/v formic acid in water and 0.1% v/v formic acid in acetonitrile; the initial gradient was 5% and increased to 35% over 12 minutes.

Analytes were detected with electrospray ionization using multiple reaction monitoring in the positive mode, based on the following precursor and product ions: SFN (mass/charge [m/z] 178, 119, 114, 72, 55), SFN–glutathione (SFN–GSH; m/z 485, 356, 308, 179, 114), SFN–Cys (m/z 299, 178, 136, 114), SFN–Cys-Gly (m/z 356, 179, 162, 136, 1,140), and SFN–*N*-acetylcysteine (SFN–NAC; m/z 341, 212, 178, 130, 114). Iberin (m/z 164, 105, 77, 72) was used as an internal standard. Acquisition and quantification were performed using Analyst software (Applied Biosystems).

### In vitro cartilage degradation assays

Cartilage explants were pretreated with 0–30 μ*M* SFN. The cytokines IL-1 or IL-1/OSM (0.5 ng/ml and 5 ng/ml, respectively) were added to induce cartilage breakdown. The treatments were refreshed every 2 days over 14 days. All treatments were performed in quadruplicate. The remaining cartilage was papain-digested overnight at 65°C. Glycosaminoglycan (GAG) and hydroxyproline were measured in the medium as described previously (20,21) and expressed as the percent release of the total.

### Animals used in the experiments

C57BL/6 mice were purchased from Harlan UK. The animal experiments were performed following ethics and statutory approval, in accordance with local policy. The mice were maintained at 21°C in standard, individually ventilated cages holding 3–6 mice per cage. The mice were fed a certified mouse diet (RM3; Special Dietary Systems) and water ad libitum. The diets were changed to AIN-93G or AIN-93G containing 0.18 or 0.6 gm/kg SFN (Research Diets) for 2 weeks prior to and following surgery, until the mice were killed.

### Destabilization of the medial meniscus (DMM) model

Ten-week-old male mice were anesthetized by inhalation of isoflurane (3% for induction and 1.5–2% for maintenance) in oxygen (1.5–2 liters/minute). All mice received a subcutaneous injection of buprenorphine (Alstoe Animal Heath) postsurgery. The mice were fully mobile within 4–5 minutes after withdrawal of isoflurane.

DMM was performed as previously described (22), and sham surgery consisted of capsulotomy only (23). The contralateral (left) knees for both procedures served as unoperated controls. OA was scored by 2 individuals (TLV and one other) in a blinded manner, using a validated histologic scoring system, as described previously (22), and results were expressed as the summed score (sum of the 3 highest total section scores for any given joint [minimum of 8 sections per joint, 80 microns apart]) (16,22,23).

### Statistical analysis

Student’s *t*-test and one-way and two-way analysis of variance (ANOVA) with Dunnett’s or Bonferroni post-test, respectively, were performed using GraphPad Prism version 5.00 for Windows. One-way ANOVA was used when testing for differences between ≥3 groups. Two-way ANOVA was used when testing for an effect of 2 factors (e.g., treatment and time).

## RESULTS

### Inhibition of cytokine-induced MMP expression in chondrocytes and synovial cells by SFN

**Figure 1:**
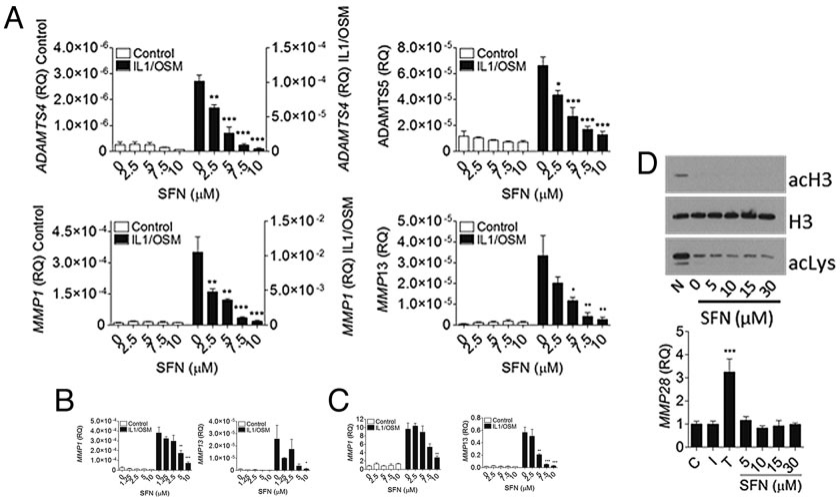
Sulforaphane (SFN) inhibits cytokine-induced metalloproteinase expression in articular joint cells. Human articular chondrocytes, fibroblast-like synoviocytes, and the SW-1353 cell line were pretreated with 0–10 μ*M* SFN and stimulated with or without interleukin-1 (IL-1; 5 ng/ml) and oncostatin M (OSM; 10 ng/ml) for 6 hours. A–C, SFN-induced inhibition of cytokine-induced *ADAMTS4*, *ADAMTS5, MMP1*, and *MMP13* mRNA expression in human articular chondrocytes (A), *MMP1* and *MMP13* mRNA expression in fibroblast-like synoviocytes (B), and *MMP1* and *MMP13* mRNA expression in SW-1353 cells (C). D, Top, Human articular chondrocyte whole cell lysates immunoblotted for acetylated histone H3 (acH3), total histone H3, and acetylated lysine (acLys). Bottom*, MMP28* mRNA expression in SW-1353 cells, as measured using quantitative reverse transcription–polymerase chain reaction. Values are the mean ± SEM (n = ≥3). RQ = relative quantification (expressed as 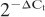); N = sodium butyrate; C = negative control; I = IL-1; T = trichostatin A. ∗ = *P* < 0.05; ∗∗ = *P* < 0.01; ∗∗∗ = *P* < 0.0001, SFN alone versus no treatment or SFN plus cytokines versus cytokines alone, by one-way analysis of variance with Dunnett’s post-test.

OA affects all of the tissue in the joint. We sought to determine quantitatively whether SFN could regulate key aggrecanases and collagenases in chondrocytes and synovial cells. SFN inhibited cytokine-induced MMP expression in human articular chondrocytes, FLS, and SW-1353 cells in a a dose-dependent manner (Figures 1A–C). In human articular chondrocytes, 2.5 μ*M* SFN significantly inhibited cytokine-induced *ADAMTS4* and *ADAMTS5* expression, 2.5 μ*M* SFN inhibited *MMP1*, and 5 μ*M* SFN inhibited *MMP13* (Figure 1A). Inhibition of gene expression in FLS or SW-1353 chondrosarcoma cells appeared to be less sensitive. In FLS, 5 μ*M* SFN inhibited *MMP1*, and 10 μ*M* inhibited *MMP13* (Figure 1B). In SW-1353 cells, 10 μ*M* SFN inhibited *MMP1*, and 5 μ*M* inhibited *MMP13* (Figure 1C). FLS and the SW-1353 cell line did not express robust levels of *ADAMTS4* or *ADAMTS5,* and therefore these were not measured. SFN did not affect the expression of *MMP2* in SW-1353 cells (data not shown).

### Effect of SFN on histone deacetylase inhibition in chondrocytes

We investigated the potential of SFN as a HDAC inhibitor in human articular chondrocytes. Whole cell lysates from chondrocytes were immunoblotted for histone H3 acetylation and general lysine acetylation. Acetylation of histone H3 or general lysine acetylation were unaltered by 0–30 μ*M* SFN (Figure 1D). Similar results were seen in SW-1353 cells (data not shown). *MMP28* is known to be regulated by HDAC inhibition in SW-1353. *MMP28* mRNA levels were not affected by SFN (Figure 1D).

### Cell viability

Cytotoxicity and activation of caspases 3/7 were measured in primary chondrocytes, FLS, and SW-1353 cells (n = 3) treated with 0–50 μ*M* SFN or 10 μ*M* staurosporine, in quadruplicate for 6 hours and 24 hours. We observed no evidence of cytotoxicity or caspase activation by SFN in these cells (data not shown).

### Effect of Nrf2 knockdown on MMP expression in chondrocytes

**Figure 2:**
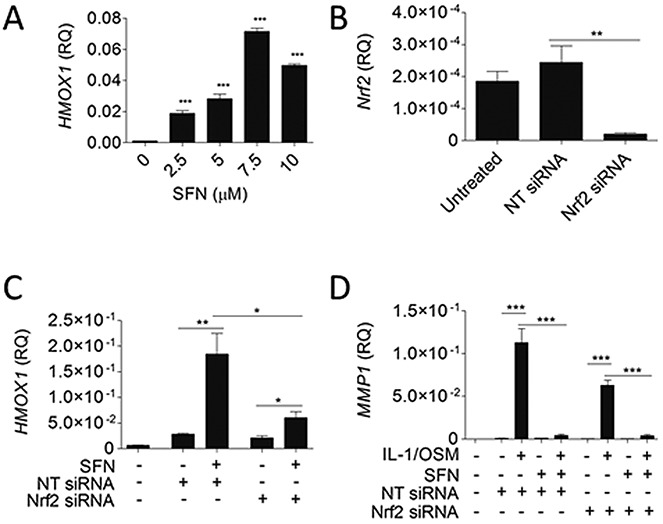
SFN does not require the NF-E2–related factor 2 (Nrf2) pathway to inhibit cytokine-induced metalloproteinase expression. Nrf2 targeting small interfering RNA (siRNA) was used to knock down Nrf2 expression in human articular chondrocytes, 24 hours prior to treatments. Human articular chondrocytes were treated with SFN (10 μ*M*) for 30 minutes prior to the addition of IL-1 (5 ng/ml) and OSM (10 ng/ml), to induce gene expression for 6 hours. Relative mRNA gene expression was measured using quantitative reverse transcription–polymerase chain reaction and normalized to 18S ribosomal RNA expression. A, SFN-induced expression of *HMOX1* mRNA. B, Silencing of *Nrf2* expression using 25 n*M* targeting siRNA in human articular chondrocytes. C, Decreased *HMOX1* expression following Nrf2 siRNA treatment. D, No impact of Nrf2 siRNA treatment on SFN-induced inhibition of cytokine-induced *MMP1* expression. Values are the mean ± SEM (n = 3). ∗ = *P* < 0.05; ∗∗ = *P* < 0.001; ∗∗∗ = *P* < 0.0001 versus 0 μ*M* SFN (A) or as indicated. NT = nontargeting siRNA control (see Figure 1 for other definitions).

The Nrf2 signaling pathway is a major mediator of SFN activity. We examined whether knockdown of Nrf2 could affect SFN-induced inhibition of MMP expression in chondrocytes. Treatment with SFN significantly induced expression of *HMOX1* (an Nrf2-regulated gene) in human articular chondrocytes in a dose-dependent manner (Figure 2A). Small interfering RNA against Nrf2 reduced Nrf2 expression in human articular chondrocytes (Figure 2B), and SFN-induced *HMOX1* expression was significantly reduced by Nrf2 siRNA compared with that induced by nontarget control (Figure 2C). IL-1/OSM–induced *MMP1* expression was inhibited with SFN treatment, and Nrf2 knockdown did not reverse the SFN inhibition of cytokine-induced *MMP1* expression (Figure 2D).

### Prolongation of MAPK activation by SFN

**Figure 3:**
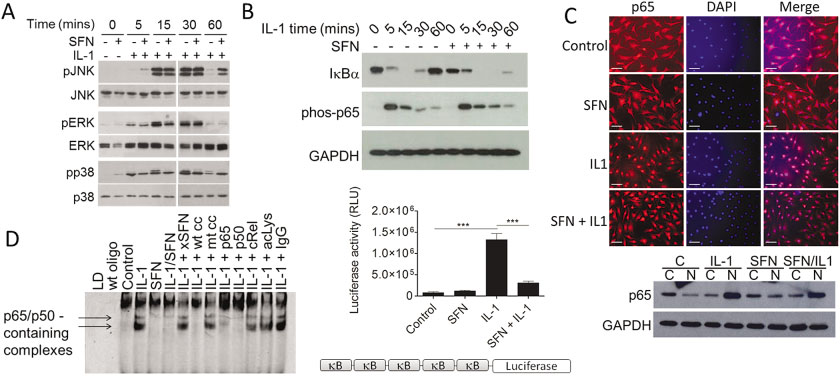
SFN regulates MAPK activation and NF-κB signaling. A, Effect of SFN on phosphorylation of JNK, ERK, and p38 MAPK in human articular chondrocytes treated with 5 ng/ml IL-1 for 0–60 minutes. B, Activation of NF-κB in human articular chondrocytes treated with IL-1 (5 ng/ml) for 0–60 minutes, with or without 10 μ*M* SFN. C, Top, Intracellular localization of p65 in human articular chondrocytes treated with IL-1 (5 ng/ml) for 45 minutes, with or without 10 μ*M* SFN. Bars = 100 μm. Bottom, Immunoblotting of cytoplasmic and nuclear fractions from human articular chondrocytes probed for p65. Translocation of p65 to the nucleus was unaffected by SFN treatment. D, Left, NF-κB binding to consensus DNA-binding sequence, as determined by electrophoretic mobility shift assay. Nuclear extracts from human articular chondrocytes were treated as described in C. Right, Inhibition of NF-κB transcriptional activation in chondrocytes by 10 μ*M* SFN, as determined by luciferase-linked κB-reporter assay. Bars show the mean ± SEM (n = 3). ∗∗∗ = *P* < 0.0001. LD = loading dye; WT = wild-type; x = exogenous; cc = competitor oligos; acLys = acetylated lysine; RLU = relative light units (see Figure 1 for other definitions).

We examined the effects of SFN on MAPK activation in primary human articular chondrocytes. SFN affected the phosphorylation kinetics of both JNK and p38 MAPK. Phosphorylation of JNK and p38 MAPK was sustained for a longer period of time with SFN pretreatment compared with IL-1 treatment alone. An unidentified higher–molecular weight band for phosphorylated p38 MAPK was seen in IL-1–treated samples, which was inhibited by pretreatment with SFN between 15 minutes and 60 minutes. SFN treatment did not affect ERK signaling in human articular chondrocytes (Figure 3A).

### Direct modulation of NF-κB signaling in human articular chondrocytes by SFN

We examined the effect of SFN on NK-κB signaling in chondrocytes. SFN treatment of human articular chondrocytes delayed the reaccumulation of IκBα following NF-κB activation by IL-1 (Figure 3B). However, phosphorylation of p65 (Ser^536^) (Figure 3B) and translocation of p65 to the nucleus (Figure 3C) were unaffected by SFN treatment in human articular chondrocytes. EMSAs for DNA binding were performed in human articular chondrocytes (Figure 3D) and SW-1353 cells (data not shown). Specific binding of the NF-κB consensus sequence was detected by the appearance of 2 bands in human articular chondrocytes when incubated with nuclear extracts from human articular chondrocytes treated with IL-1 for 45 minutes. These 2 bands were blocked with the addition of anti-p65 or anti–p50 NF-κB antibodies, respectively. These bands could also be competed with unlabeled wild-type but not mutant oligonucleotide, demonstrating specificity. Nuclear extracts from human articular chondrocytes pretreated with SFN prior to treatment with IL-1 showed substantially diminished binding of the upper band and complete abrogation of binding to the lower band. Acetylated proteins in the upper of the 2 bands containing p65 and p50 complexes were detected, whereas the lower band remained largely unaffected by the addition of pan–acetylated lysine antibody. The addition of 10 μ*M* exogenous SFN directly into the DNA-binding reaction did not affect NF-κB binding (Figure 3D). A luciferase-linked κB-promoter reporter assay showed that pretreatment with 10 μ*M* SFN significantly inhibited IL-1–induced NF-κB signaling (*P* < 0.0001) (Figure 3D).

### Effect of SFN on the expression kinetics of a panel of known NF-κB–responsive genes in human articular chondrocytes

**Figure 4:**
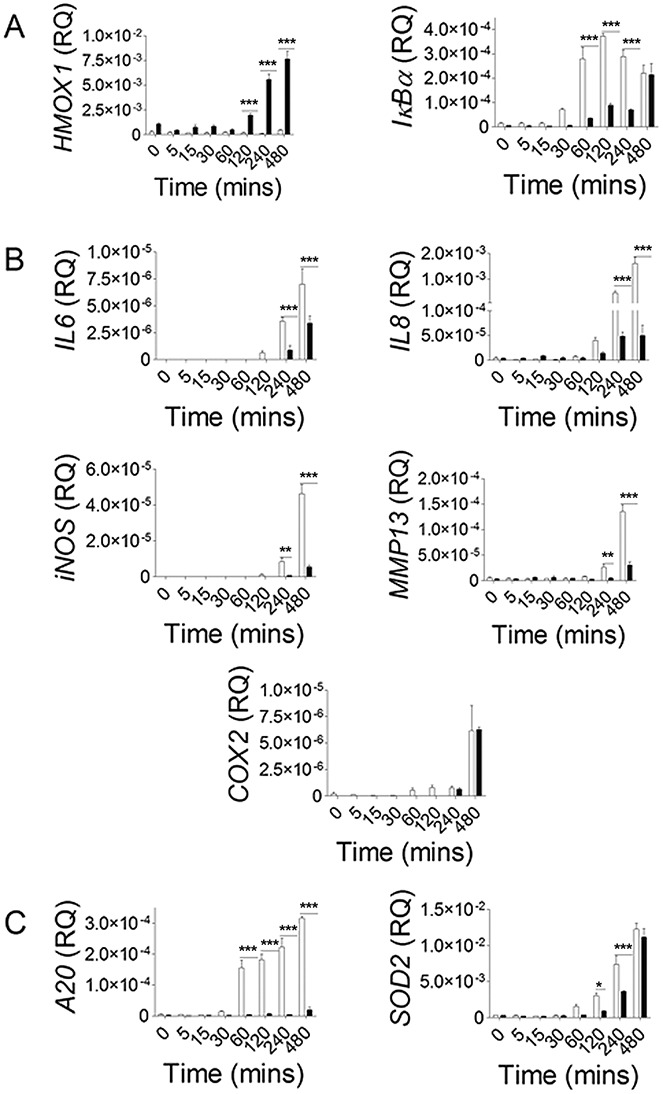
SFN treatment inhibits expression of known NF-κB–responsive genes. Human articular chondrocytes were treated with 5 ng/ml IL-1 for 0–8 hours, with (▪) or without (□) 10 μ*M* SFN. Gene expression was measured using real-time quantitative reverse transcription–polymerase chain reaction and normalized to 18S ribosomal RNA expression. A, Expression of *HMOX1* and *IκBα*. B, Expression of the inflammatory genes *IL6, IL8*, *iNOS*, *MMP13*, and *COX2*. C, Expression of the cytoprotective genes *A20* and *SOD2*. Values are the mean ± SEM (n = 3). ∗ = *P* < 0.05; ∗∗ = *P* < 0.01; ∗∗∗ = *P* < 0.001, by two-way analysis of variance with Bonferroni post-test. See Figure 1 for other definitions.

The regulation of NF-κB signaling by SFN was confirmed by investigating the expression of NF-κB–responsive genes in cultured human articular chondrocytes. *HMOX1* was measured as an example of specific SFN activity in human articular chondrocytes not influenced by IL-1 treatment (Figure 4A). *HMOX1* was highly expressed with SFN treatment, beginning at 2 hours (*P* < 0.001). Expression of *IκBα* was significantly inhibited from 60 minutes to 4 hours, but *IκBα* mRNA levels, with and without SFN treatment, were equal by 8 hours (Figure 4A). SFN treatment significantly inhibited the transcription of inflammatory NF-κB–responsive genes induced by IL-1 (*IL6*, *IL8, iNOS,* and *MMP13*) at 4 and 8 hours (Figure 4B). *COX2* mRNA expression was not significantly affected. Expression of the cytoprotective gene *A20* was completely abolished with SFN treatment at 1–8 hours (Figure 4C). Expression of *SOD2* was more similar to that of *IκBα*, with inhibition between 2 and 4 hours and mRNA levels equal by 8 hours (Figure 4C).

### Accumulation of SFN metabolites in chondrocytes

**Figure 5:**
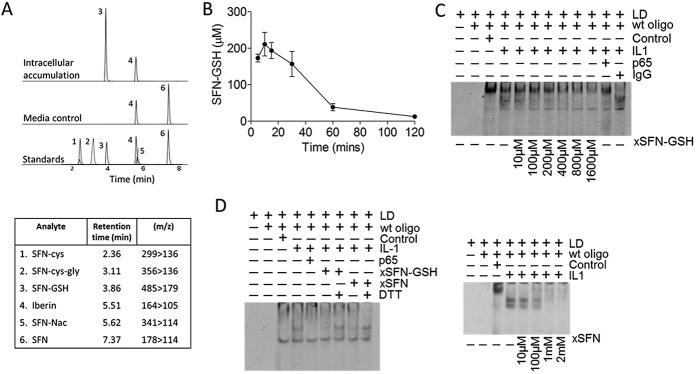
Sulforaphane (SFN) accumulates primarily as SFN–glutathione (SFN–GSH) in chondrocytes and can directly inhibit NF-κB binding to the consensus DNA sequence. Cell-free medium (control) and primary chondrocytes were treated with 10 μ*M* SFN for 0–2 hours, with iberin used as an internal control. A, Expression of SFN–Cys, SFN–Cys-Gly, SFN–GSH, iberin, SFN–*N*-acetylcysteine (SFN–NAC), and SFN, as determined by high-performance liquid chromatography tandem mass spectrometry (HPLC-MS/MS). B, Intracellular accumulation of SFN–GSH in primary chondrocytes, as quantified by HPLC-MS/MS. Values are the mean ± SEM. C, Titration of exogenous SFN–GSH (top) or exogenous SFN (bottom) into DNA-binding reaction mixtures. The binding reaction mixtures contained nuclear extracts from human articular chondrocytes stimulated with interleukin-1 (IL-1; 5 ng/ml) for 45 minutes and the NF-κB consensus DNA sequence. D, Preincubation of exogenous SFN–GSH or exogenous SFN with the reducing agent dithiothreitol (DTT) for 15 minutes rescued inhibition of nuclear extract/DNA binding (final concentrations 250 μ*M* exogenous SFN–GSH or exogenous SFN and 17.9 m*M* DTT). m/z = mass/charge (see Figure 3 for other definitions).

The 10-μ*M* SFN treatment applied to chondrocytes in culture was not sufficient to inhibit the direct NF-κB/DNA binding reaction on EMSA (Figure 3D). We determined the intracellular concentration of SFN and its metabolites in primary chondrocytes and SW-1353 cells. The accumulation of SFN and its metabolites was characterized within chondrocytes following the addition of 10 μ*M* SFN to culture medium. Parallel incubations of SFN were conducted in cell-free medium (DMEM) to control for degradation of media matrix. SFN–Cys, SFN–Cys-Gly, SFN–GSH, SFN–NAC, and SFN were detected in primary chondrocytes and/or culture medium (Figure 5A) and SW-1353 cells (results not shown). Primary chondrocytes treated with 10 μ*M* SFN for 0–2 hours showed a peak accumulation within 10–15 minutes, at 150–275 μ*M* (Figure 5B). SW-1353 cell accumulation occurred within 1 hour, at 1–1.6 m*M* (results not shown). In both cell types, the predominant intracellular form of SFN was SFN–GSH. Free SFN was not detected intracellularly. Exogenous SFN–GSH or exogenous SFN was titrated into nuclear extract/DNA–binding reactions. Both SFN–GSH and SFN (Figure 5C) diminished NF-κB–DNA binding. Incubation of SFN–GSH or SFN with DTT prior to being added to the DNA-binding reaction rescued NF-κB/DNA binding (Figure 5D).

### Chondroprotective effect of SFN in vitro and in vivo

**Figure 6:**
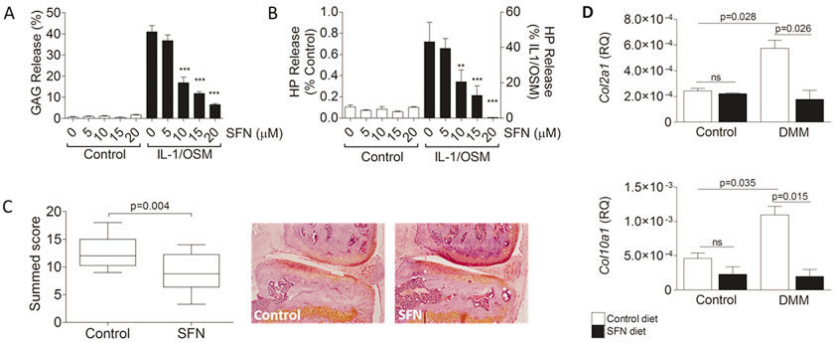
SFN inhibits cartilage destruction in a bovine explant model and protects against surgically induced osteoarthritis in mice in vivo. A and B, Release of glycosaminoglycan (GAG) (A) and hydroxyproline (HP) (B) into culture medium. Bovine nasal cartilage explants were treated with 0–20 μ*M* SFN, with or without IL-1 (0.5 ng/ml) and OSM (5 ng/ml), for 14 days. Values are the mean ± SEM (n = 4). ∗∗ = *P* < 0.001; ∗∗∗ = *P* < 0.0001 versus no treatment. C, Left, Summed scores of each histologic section obtained through the joints of C57BL/6 mice that were fed a control diet or a diet containing 0.18 gm/kg SFN ad libitum, 2 weeks prior to and following destabilization of the medial meniscus (DMM). Data are shown as box plots. Each box represents the 25th to 75th percentiles. Lines inside the boxes represent the median. Lines outside the boxes represent the 10th and the 90th percentiles. Right, Representative Safranin O–stained histologic sections (8 μm) obtained from the medial joint compartment of mice fed a control diet and those fed an SFN-rich diet. Original magnification × 40. D, Expression of *Col2a1* mRNA (top) and *Col10a1* mRNA (bottom) in the joints of unoperated control mice (n = 13) and mice that underwent DMM (n = 17), that were fed a control diet or an SFN-rich diet (0.6 gm/kg), as determined by quantitative reverse transcription–polymerase chain reaction. Values are the mean ± SEM (n = 3). See Figure 1 for other definitions. Color figure can be viewed in the online issue, which is available at http://onlinelibrary.wiley.com/doi/10.1002/art.38133/abstract.

We used a short-term in vitro bovine nasal cartilage model of cartilage destruction to investigate the chondroprotective activity of SFN in tissue (24). On day 2, IL-1/OSM–induced GAG release was significantly repressed by 10 μ*M* SFN and was further repressed, in a dose-dependent manner, by 15 and 20 μ*M* SFN (Figure 6A). IL-1/OSM–induced hydroxyproline release was also significantly inhibited by 10 μ*M* SFN on day 11 (Figure 6B).

We sought to confirm the data in an in vivo murine model of cartilage destruction. Among mice that underwent DMM, those fed an SFN-rich diet (3 μmoles/day) had significantly reduced cartilage destruction at 12 weeks compared with those fed a control diet (*P* = 0.004) (Figure 6C). There was no significant alteration in the body weight of mice across the experiment (data not shown). Whole-genome microarray analysis of mouse whole-joint tissue at 24 hours and the day 7 postoperative time points identified a number of regulated mRNAs. Candidate genes with expression changes of ±1.5-fold were validated using qRT-PCR. Procollagen, *Col2a1*, and *Col10a1* mRNA levels were significantly increased in the destabilized joints compared with controls (*P* = 0.028 and *P* = 0.035, respectively) on day 7. Increased expression of *Col2a1* and *Col10a1* mRNA was not observed in mice fed an SFN-rich diet (*P* = 0.026 and *P* = 0.015, respectively). The SFN diet had no effect on basal *Col2a1* and *Col10a1* expression (Figure 6D).

## DISCUSSION

The regulation of catabolic factors in more than one joint tissue is important in OA (25). Our data for primary chondrocytes demonstrate that SFN dose-dependently inhibited IL-1/OSM–induced expression of collagenases (*MMP1* and *MMP13*) and aggrecanases (*ADAMTS4* and *ADAMTS5*). These findings support an earlier report that SFN inhibited IL-1/TNFα–induced collagenase expression (10). We also observed SFN-induced inhibition of collagenase expression in OA FLS.

In rheumatoid synovial cells, SFN was reported to inhibit hyperplasia and induce apoptosis (12), although SFN was administered at high doses in vivo via intraperitoneal injection. Our measurements in primary human articular chondrocytes, SW-1353 cells, and FLS (data not shown) are consistent with previous reports (10,26) that SFN is not cytotoxic at concentrations attained in the plasma from dietary intake (27).

We initially hypothesized that SFN was chondroprotective via the inhibition of HDAC activity, because SFN has been reported to be a weak HDAC inhibitor in other cell types (17,28). We did not observe any evidence to support SFN as an inhibitor of HDAC in chondrocytes, either directly or as an inducer of *MMP28*.

SFN is a potent inducer of the transcription factor Nrf2, and Nrf2 is an important mediator of SFN activity in several cell types (6). Interestingly, Guillén et al showed up-regulation of *HMOX1* (an Nrf2-regulated gene) by cobalt protoporphyrin IX in chondrocytes coincided with a reduction in MMP-1 and MMP-13 expression and an increase in type II collagen and aggrecan expression (29). Our knockdown of Nrf2 showed that SFN remained able to inhibit cytokine-induced *MMP1* expression, independently of Nrf2; however, activation of Nrf2 by SFN will likely contribute chondroprotective effects through the induction of cytoprotective genes, including *HMOX1* (30–33).

Regulation of NF-κB signaling by SFN has been reported. Inhibition of IκB phosphorylation and/or degradation, IKK phosphorylation, and NF-κB nuclear translocation by SFN are described in various cell types, including chondrocytes (11,34–36). We did not observe any evidence demonstrating that SFN regulated these mechanisms in primary human articular chondrocytes. Moreover, our studies showed that SFN treatment affected the reaccumulation kinetics of IκBα, likely due to postactivation inhibition of NF-κB (37). This was supported by the complete ablation of *IκBα* and *A20* expression required for signaling through the negative feedback loop of NF-κB.

In accordance with our data, Heiss et al (37) reported that SFN inhibited NF-κB/DNA binding and did not affect IκB degradation or NF-κB nuclear translocation in response to lipopolysaccharide. Although the *trans*-activating inhibitory activity of NF-κB by SFN cannot be ruled out (38), our evidence suggests that SFN-induced inhibition of NF-κB signaling is generic, given that the expression of both proinflammatory and cytoprotective genes regulated by NF-κB was decreased, albeit with distinct gene-dependent kinetics.

NF-κB is a known redox-sensitive transcription factor requiring a reducing environment for DNA binding. Heiss et al proposed that SFN could inhibit NF-κB directly by forming dithiocarbamates with NF-κB Cys residues or indirectly via inhibiting, e.g., thioredoxin/thioredoxin reductase, thereby modulating redox potential (37,38). This proposition was supported by the observation that a reducing agent reversed the SFN-induced inhibition of transcription factor binding (39), although it is in opposition with thioredoxin being an Nrf2-responsive gene (40). SFN has been reported to accumulate intracellularly via conjugation with glutathione in a cell-dependent manner (41,42). We show that SFN at high concentrations (between 150–275 μ*M*) accumulates primarily as SFN–GSH in primary human chondrocytes, and this concentration of SFN–GSH can directly inhibit NF-κB/DNA binding to the NF-κB consensus sequence.

Kim et al previously reported that SFN inhibited cytokine-induced NF-κB DNA binding and JNK activation (10). Our results corroborate these data in part, although we did not observe consistent inhibition of JNK activation in primary human articular chondrocytes across donors. We did observe that compared with IL-1 alone, the addition of SFN consistently prolonged JNK and p38 MAPK phosphorylation. Extended MAPK activation has been attributed to a lack of NF-κB feedback and TNF-induced accumulation of reactive oxygen species (43). In other cell types, this leads to cell death, which was not observed in our models. It has been reported that direct phosphorylation of Nrf2 by the p38δ MAPK isoform promoted the association between Nrf2 and Kelch-like ECH-associated protein 1 proteins and subsequent inhibition of Nrf2 activity (44). Our data suggest that SFN can inhibit an isoform of p38 MAPK; if this is confirmed, it would be consistent with the prosurvival/antiapoptotic properties of SFN reported in chondrocytes as p38 dependent (26). It remains unclear whether the effects of SFN on MAPK signaling are additive, synergistic, or a result of altered NF-κB signaling.

The protective effect of SFN at the tissue level in bovine nasal cartilage explants supports recent findings by Kim et al (11). We now show that SFN obtained from the diet is protective against OA in vivo. Because mice feed by grazing rather than by consuming a daily meal, there is no easy means with which to compare mice with humans. However, the dosage chosen (3 μmoles/day) gives an overall delivery that is deemed to be the high end of physiologic (∼2.3–7.4 μmoles/liter in plasma in humans) (27,45). A whole-genome array analysis of mouse whole knee joint tissue uncovered a number of regulated genes. Full analysis of these data is beyond the scope of this study; however, regulation of *Col2a1* and *Col10a1* was of particular interest. The increased expression of *Col2a1* and *Col10a1* on day 7 after DMM was performed may represent facets of both protective and injury responses, respectively.

*COL2A1* expression is regulated cooperatively by SOX9, L-SOX5, and SOX6 (46–48), and it is reported that NF-κB p65 activity may precede that of SOX9 to initiate chondrogenic differentiation (49). More recently, p65 was shown to specifically bind the *COL2A1* intronic enhancer to regulate *COL2A1* as well as *SOX9* expression (50). Hypoxia-inducible factor 2α1, encoded by *EPAS1*, has been reported as the most potent transactivator of *COL10A1* expression in cultured chondrocytes, and *EPAS1* itself is strongly induced by NF-κB p65 activity (51). Mice fed the SFN-rich diet did not show increased expression of *Col2a1* or *Col10a1* mRNA in response to DMM surgery, potentially because of a dampened NF-κB response in vivo. Our data therefore show that a high-glucosinolate diet may be a useful measure either to prevent or to slow the progression of OA.

## AUTHOR CONTRIBUTIONS

All authors were involved in drafting the article or revising it critically for important intellectual content, and all authors approved the final version to be published. Dr. Clark had full access to all of the data in the study and takes responsibility for the integrity of the data and the accuracy of the data analysis.

**Study conception and design.** Davidson, Kay, Culley, Bao, Clark.

**Acquisition of data.** Davidson, Jupp, de Ferrars, Norton, Driscoll, Vincent, Donell.

**Analysis and interpretation of data.** Davidson, Jupp, Kay, Culley, Bao, Clark.
